# GPER expressed on microglia mediates the anti‐inflammatory effect of estradiol in ischemic stroke

**DOI:** 10.1002/brb3.449

**Published:** 2016-03-22

**Authors:** Tian‐Zhi Zhao, Qian Ding, Jun Hu, Shi‐Ming He, Fei Shi, Lian‐Ting Ma

**Affiliations:** ^1^ Department of NeurosurgeryWuhan General Hospital of Guangzhou Military Command of Chinese PLAWuhan430070Hubei ProvinceChina; ^2^ Department of NeurosurgeryTangdu HospitalFourth Military Medical UniversityNo. 569 Xinsi RoadBaqiao DistrictXi'an710038Shanxi ProvinceChina; ^3^ Department of AnesthesiologyTangdu HospitalFourth Military Medical UniversityNo. 569 Xinsi RoadBaqiao DistrictXi'an710038Shanxi ProvinceChina; ^4^ Department of NeurologyChinese PLA No. 451 HospitalXi'an710054Shanxi ProvinceChina; ^5^ Department of Aerospace BiodynamicsFourth Military Medical UniversityXi'an710032Shanxi ProvinceChina

**Keywords:** GPER, inflammation, ischemic stroke, microglia, estradiol

## Abstract

**Background:**

Stroke could lead to serious morbidity, of which ischemic stroke counts for majority of the cases. Inflammation plays an important role in the pathogenesis of ischemic stroke, thus drugs targeting inflammation could be potentially neuroprotective. Estradiol was shown to be neuroprotective as well as anti‐inflammatory in animal models of ischemic stroke with unclear mechanism. We hypothesize that the anti‐inflammatory and neuroprotective effect of estradiol is mediated by the estradiol receptor G protein‐coupled estrogen receptor 1 (GPER) expressed on microglia.

**Methods:**

We have generated the rat global cerebral ischemic model and the primary microglia culture to study the neuroprotective and anti‐inflammatory effect of estradiol. We have further used pharmacological methods and siRNA knockdown approach to study the underlying mechanism.

**Results:**

We found that estradiol reduced the level of proinflammatory cytokines including IL‐1*β* and TNF‐*α*, both in vivo and in vitro. We also found that the specific GPER agonist G1 could reduce the level of IL‐1*β* (*P* = 0 *P* = 0.0017, one‐way ANOVA and post hoc test) and TNF‐*α* (*P* < 0.0001) in the primary microglia culture. Moreover, the specific GPER antagonist G15 was able to abolish the anti‐inflammatory effect of estradiol. Estradiol failed to reduce the level of IL‐1*β* (*P* = 0.4973, unpaired Student's *t*‐test) and TNF‐*α* (*P* = 0.1627) when GPER was knocked down.

**Conclusions:**

Our studies have suggested that GPER expressed on microglia mediated the anti‐inflammatory effect of estradiol after ischemic stroke. Our studies could potentially help to develop more specific drugs to manage inflammation postischemic stroke.

## Introduction

Stroke is one of the leading causes of permanent morbidity and mortality in the world (Donnan et al. 2008). Ischemic stroke, which counts for 60~80% of total cases, is characterized by interrupted cerebral blood flow leading to the neuron death in the brain (Donnan et al. 2008). Current therapies of thrombolysis and thrombectomy is effective, only if patients were treated promptly (Meyers et al. 2011). It is tempting to develop additional pharmacological interventions that do not pose a limitation of time. Inflammation, which includes activation of local microglia and recruitment of other immune cells from the blood, as well as production of proinflammatory cytokines, plays a vital role in the pathogenesis of ischemic stroke (Jin et al. 2010). Suppressing inflammation could reduce the infarct size and alleviating the neuronal damage (Wang 2005; Yilmaz and Granger 2008). However, due to the multifaceted roles of inflammatory mediators, the application of anti‐inflammatory approaches is less successful in clinical than in experimental animals (Enlimomab Acute Stroke Trial 2001; Becker 2002; Amantea et al. 2014). Further research about the inflammation during ischemic stroke is needed to gain a more comprehensive understanding and a smarter application.

Estradiol (*β*‐estradiol or E2), which is a steroid hormone and also the major biologically active estrogen hormone, has been shown to have neuroprotective function broadly in animal models of ischemic stroke (Dubal et al. 1998; Toung et al. 1998; Hurn and Macrae 2000). The neuroprotective effect of estradiol is possibly mediated by ERs (estrogen receptors), which includes both classical ERs and nonclassical ERs (Arevalo et al. 2015). G protein‐coupled estrogen receptor 1, or GPER, is a nonclassic ER that was discovered recently to be responsible for the nongenomic or rapid cellular signaling of estradiol (Revankar et al. 2005). GPER is expressed broadly in neurons and microglia in the cortex; however, the neuroprotective function of GPER in ischemic stroke has not yet been thoroughly studied (Brailoiu et al. 2007; Hazell et al. 2009). We hypothesize that GPER mediates the neuroprotective function of estradiol through suppressing postischemic inflammation. In this study, we found that GPER expressed on microglia mediated estradiol‐induced inhibition of inflammation, which might contribute to the neuroprotective function of estradiol. Our studies would improve our understanding of regulating inflammation after ischemic stroke and help develop new treatments for stroke.

## Materials and Methods

### Animal

**Figure 1:**
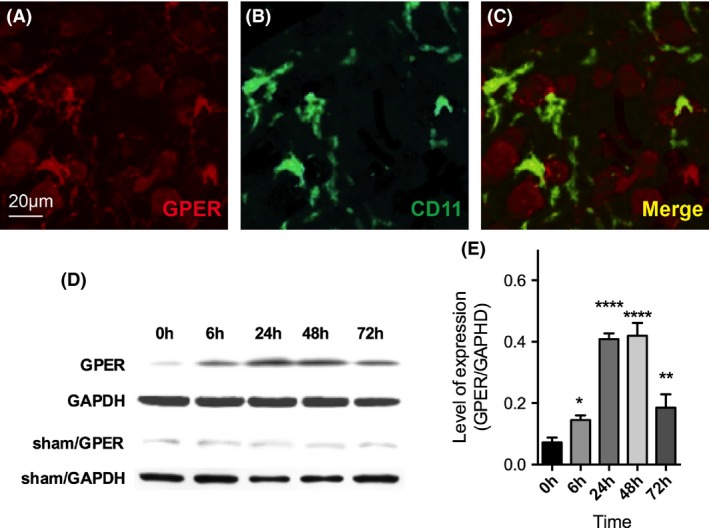
GPER was expressed on forebrain microglia and hippocampal region after cerebral ischemia. (A) GPER (red) immunostaining in the forebrain motor cortex; (B) CD11b (green) immunostaining in the forebrain motor cortex; (C) merge of A and B. (D) western blot detection of GPER in ischemic rats and sham rats from 6 to 72 h. (E) the quantification of GPER expression in ischemic rats. The expression of GPER started to increase at 6 h (*n* = 3, *P* = 0.0439, one‐way ANOVA and post hoc test) and reached peak at 48 h (*P* < 0.0001). Data were presented as mean + SD. *P < 0.05, **P < 0.01, ****P < 0.0001.

**Figure 2:**
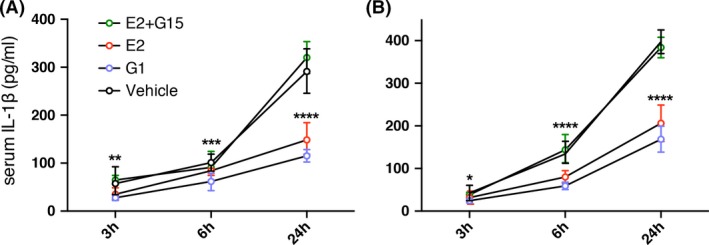
Injection of *β*‐estradiol (E2) and GPER‐specific agonist G1 reduced serum IL‐1*β* and TNF‐*α* concentrations. (A) serum concentration of IL‐1*β* in 3, 6, and 24 h in postischemic rats in the control group (DMSO, black circle), *β*‐estradiol injected group (Red circle), G1‐injected group (Purple circle), and *β*‐Estradiol + G15injected group (Green circle); There is significant difference between E2 or G1 injection group with control group (E2, 24 h, *P* < 0.0001; G1, 3 h, *P* = 0.0036, 6 h, *P* = 0.0002, 24 h, *P* < 0.0001). There is no significant difference between E2 + G15 group with the control group (3 h, *P* = 0.8048; 6 h, *P* = 0.6572, 24 h, *P* = 0.1682). (B) serum concentration of TNF‐*α* postischemic rats in the control group (saline, black circle), *β*‐estradiol injection group (Red circle), and G1 injection group (Purple circle). There is significant difference between E2 or G1 injection group with control group (E2, 6 h, 24 h, *P* < 0.0001; G1, 3 h, *P* = 0.0420, 6 h & 24 h, *P* < 0.0001). There is no significant difference between E2 + G15 group with the control group (3 h, *P* = 0.9456, 6 h, *P* = 0.5393, 24 h, *P* = 0.2636). Data were presented as mean ± SD. *P < 0.05, **P < 0.01, ***P < 0.001, ****P < 0.0001.

Adult female Sprague–Dawley rats weighing 250–300 g were used in generating global cerebral ischemia model and additional further tests. This study was carried out in strict accordance with the recommendations in the Guide for the Care and Use of Laboratory Animals of the National Institutes of Health (1986). The protocol was approved by the Committee on the Ethics of Animal Experiments of Wuhan General Hospital of Guangzhou Military Command of Chinese PLA (20140627). All surgeries were performed under sodium pentobarbital anesthesia, and all efforts were made to minimize suffering. For expression experiments (Fig. 1), each experiment was repeated for three rats, and in total, nine rats were used. For in vivo experiments (Fig. 2), five rats were used for each time point and in total, 60 rats were used in this experiment. Besides these 60 rats, three rats died during the experimental procedure and they were not used in further analysis. For in vitro experiments, 58 neonatal rats (P2) were used in total. On an average, one rat yields 3~4 microglia culture plates (2 × 10^5^ cells per plate).In each group, 10 plates were used, and in total, 180 plates were used. Besides these 58 rats, data of 12 rats were excluded from the analysis because the microglia primary cultures were not maintained successfully (due to contamination, etc.). Blind tests were performed, in which the drug names were masked from the experimenters, and the experiment group names were masked from the data analysts according to STAIR guidelines.

### Global cerebral ischemia model

Female rats were anesthetized with 40 mg/kg pentobarbital sodium (i.p.) and subsequently placed in prone position on an automated heating pad (Kent Scientific, Torrington, CT) to maintain the body temperature of 37°C. An incision of 2 cm was made on the midline of the dorsal cervical surface to expose the alar foramina of the first cervical vertebrae of both sides. We used dissection microscope (OLYMPUS, Tokyo, Japan) to expose vertebral arteries that travel below the alar foramina and insert the electrocautery needle (Bowie Electrocautery, Douglassville, TX) to alar foramina of both sides to cauterize vertebral arteries. After arterial occlusion, the wound was sutured and the rats were returned to their cages. After 24 h, rats were reanesthetized using the same method and electrodes were mounted underneath the scalp on the parietal region of left hemisphere to monitor the EEG (electroencephalogram). Rats were then put in supine position and an incision of 2 cm was made on the midline of the ventral cervical surface to expose common carotid arteries. After isolating common carotid arteries, an arterial clasp was placed around each common carotid artery. Both clasps were closed for 15 min and EEG was monitored as the criterion of the global cerebral ischemia. Then, both clasps were opened after 15 min to restore the blood supply and closed the wound.

### Immunohistochemistry & Nissl staining

Rats were anesthetized with 100 mg/kg pentobarbital sodium (i.p.) and subsequently perfused with cold PBS followed by 4% PFA (paraformaldehyde). After perfusion, the forebrain was dissected. Later, the motor cortex region and hippocampus were identified, postfixed in 4% PFA for 1 h, and cryoprotected in sucrose overnight. Tissue was later cut into blocks, embedded in OCT and frozen sectioned into 20 *μ*m slices. Double immunostaining of CD11 and GPER was performed in motor cortex slices using primary antibodies of rabbit anti‐GPR30 (Abcam, 1:300, Cambridge, MA) and mouse anti‐CD11b (Abcam, 1:5000). Nissl staining was performed on hippocampal slices using cresyl violet following the previously described method in Paxinos and Watson 1986 (Paxinos and Watson 1986).

### Chemicals & drug delivery

*β*‐Estradiol was obtained from Sigma (St. Louis, MO). The GPER‐specific agonist G1 and antagonist G15 were obtained from Tocris Bioscience (Shanghai, China). *β*‐Estradiol (0.1 *μ*g/kg body weight), G1 (0.5 *μ*g/kg body weight), and G15 (4.7 *μ*g/kg body weight) were diluted in 50 *μ*L DMSO and injected intravenously 15 min after ischemia was induced. For knocking down GPER1, we generated the GPER antisense oligonucleotide 5′‐TTGGGAAGTCACATCCAT‐3′ (Eurogenetec Ait, Singapore), as previously described by Lu et al. 2009 (Lu et al. 2009). For the sham group, we generated a random oligonucleotide of 5′‐GTATATCCCTGGAAACTA‐3′. We transfected the two siRNAs into microglia primary culture using Lipofectamine 2000 (Invitrogen, Pleasanton, CA) following the manufacturer's instructions.

### Primary culture of microglia

We dissected and cleaned the neonatal rat (P2) brain tissue, after which we suspended cells from brain tissue and centrifuged to obtain the mixed cell culture. We then purified microglia from the mixed cell culture following protocol described in Tamashiro et al. 2012 (Tamashiro et al. 2012). Three plates of microglia primary culture were generated for one rat.

### Cytokines release assay

Rat serum was harvested from the whole rat blood and culture supernatants were removed from microglia primary culture by centrifuging. IL‐1*β* and TNF‐*α* concentrations were measured by ELISA kit (BD Pharmingen, San Diego, CA) following the manufacturer's manual.

### Western blotting analysis

Cells from the microglia primary culture or tissue of rat hippocampus were homogenized and samples were loaded to run SDS‐polyacrylamide gel electrophoresis (10%). After transfer, the membrane was incubated in rabbit anti‐GPR30 monoclonal antibody (Abcam, 1:200) or rabbit anti‐beta tubulin polyclonal antibody (Abcam, 1:500). After washing, the membranes were incubated with a HRP (horseradish peroxidase) conjugated secondary antibody goat anti‐rabbit IgG (Abcam, 1:2000), and later exposed by the chemiluminescence kit (Abcam) following manufacturer's instructions.

### Statistics

**Figure 3:**
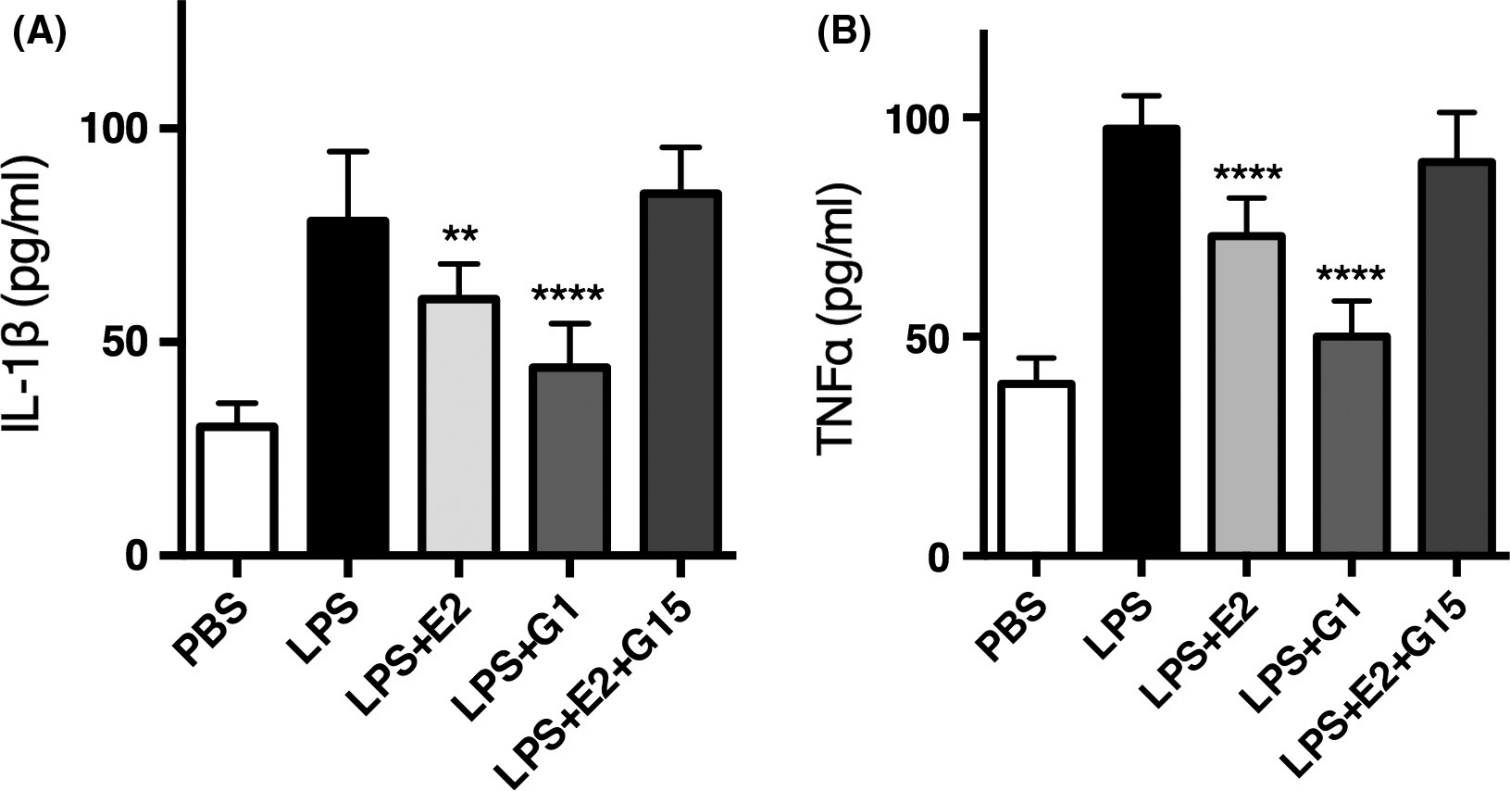
G1 and *β*‐estradiol were able to reduce the secretion of IL‐1*β* and TNF‐*α* in primary microglia culture. (A) LPS‐induced IL‐1*β* secretion was suppressed by *β*‐estradiol (*n* = 10, *P* = 0.0017, one‐way ANOVA and post hoc test, same as below) and G1 (*n* = 10, *P* < 0.0001). G15 abolished the inhibitory effect of *β*‐estradiol (*n* = 10, *P* = 0.4973). (B) LPS‐induced TNF‐*α* secretion was suppressed by *β*‐estradiol (*n* = 10, *P* < 0.0001) and G1 (*n* = 10, *P* < 0.0001). G15 abolished the inhibitory effect of *β*‐estradiol (*n* = 10, *P* = 0.1627). Data were presented as mean + SD. **P < 0.01, ****P < 0.0001.

**Figure 4:**
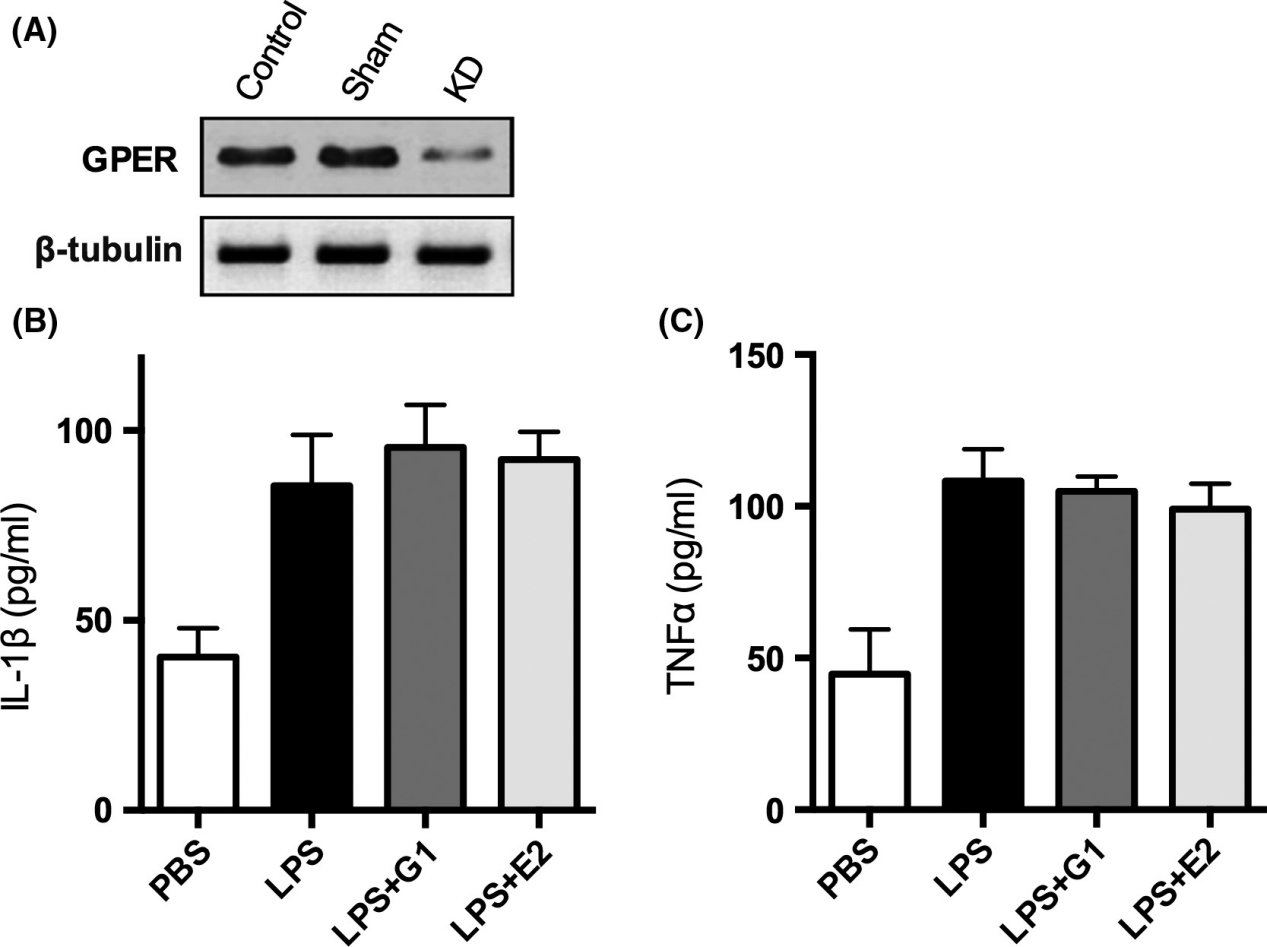
Knockdown of GPER abolished the anti‐inflammatory effect of *β*‐estradiol in primary microglia culture. (A) reduced expression of GPER in knockdown group, but not in control (untransfected) and sham (random oligonucleotide) groups. G1 and *β*‐estradiol were unable to significantly reduce the secretion of IL‐1*β* (B) and TNF‐*α* (C) when the expression of GPER microglia was knocked down (*n* = 10, IL‐1*β*,* P* = 0.2330, TNF‐*α*,* P* = 0.1017, one‐way ANOVA). Data were presented as mean + SD.

Statistical analyses were performed with Prism (Graph‐Pad Software Inc., San Diego, CA) software. Data was presented as mean ± SD. In Figure 2, two‐way analysis of variance (ANOVA) and Sidak's multiple comparison tests were performed to evaluate the significance of the data and calculate the adjusted *P* value. In Figures 1, 3, one‐way ANOVA and post hoc test were performed to evaluate the significance of the data and calculate the adjusted *P* value. In Figure 4, one‐way ANOVA test was performed to evaluate the significance of the data and calculate the *P* value.

## Results

### The rat model of global cerebral ischemia

**Figure 5:**
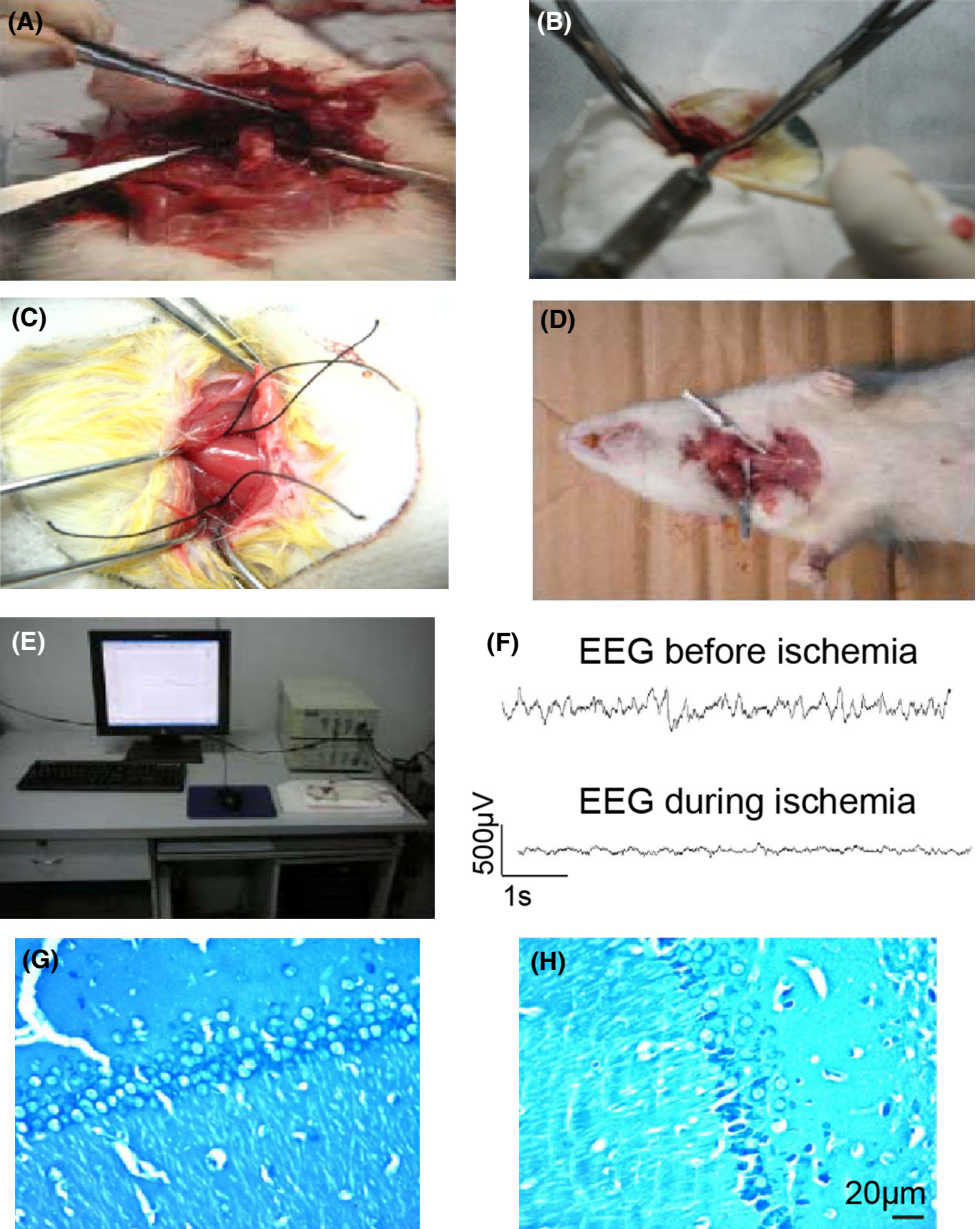
(A, B) the rat model of global cerebral ischemia; (C, D) demonstration of clasping common carotid arteries; (E, F) EEG recording before and during inducing cerebral ischemia; (G, H) Nissl staining of the hippocampal neurons before and 3 days after inducing cerebral ischemia.

To study the function of GPER in ischemic stroke, it is fundamental to create the animal model of cerebral ischemia. We have used the “four vessel occlusion” (4VO) approach to generate the rat model of global cerebral ischemia (Pulsinelli and Brierley 1979). We have recorded the electroencephalography (EEG) of both hemispheres of rats before and during the induced global cerebral ischemia and found that the spontaneous EEG firings became flat when we clasped common carotid arteries, with representative traces shown in Figure 5F. To further validate the cerebral ischemia model, we performed Nissl staining 3 days postischemia to examine the survival of hippocampal neurons, which were reportedly most vulnerable to this approach of generating brain ischemia (Pulsinelli and Brierley 1979). We found that when compared with normal rat hippocampal tissue, ischemic hippocampal slice had showed increased number of condensed nucleus, which was consistent with the anticipated cell death after ischemia. The representative images were shown in Figure 5G and H. The EGG and Nissl staining were both repeated three times. To summarize, we have established the rat global cerebral ischemia model to assist us to further study the molecular function implicated in ischemia.

### GPER was expressed on forebrain microglia and hippocampal region after cerebral ischemia

After establishing the rat model of global cerebral ischemia, we further examined the expression of GPER in the forebrain cortex 24 h after ischemia was induced. Previous paper using immunohistochemistry had suggested a high‐expression level of GPER in the forebrain including the motor and somatosensory cortex, hypothalamus, and hippocampus. Our results were consistent with previous studies that we found a high expression of GPER in the motor cortex of forebrain (Fig. 1A) in postischemic rats . Especially, a fraction of the GPER staining overlapped with the microglia marker CD11b staining, which indicated the expression of GPER on microglia (Fig. 1B and C). We also examined the GPER expression in the hippocampal region in ischemic rats by western blot (Fig. 1D and E). We found that after global ischemia, hippocampal GPER expression started to increase at 6 h (*n* = 3, *P* = 0.0439, one‐way ANOVA and post hoc test). The expression reached peak at 48 h (*P* < 0.0001) and started to decrease after 72 h (*P* = 00033). In the meantime, GPER expression in sham rats remained stable. The expression timing of GPER correlated well with the inflammatory process postischemia. Collectively, we have confirmed the expression of GPER after global ischemia.

### *β*‐Estradiol has inhibitory effect on inflammation postischemia and this effect was mimicked by G1 and blocked by G15

After detecting GPER expression on the activated microglia, we further investigated the underlying function of GPER after cerebral ischemia. *β*‐Estradiol (E2) was shown to present inhibitory effect on the inflammation process postischemia, and the underlying mechanism remained elusive. We hypothesized that the inhibitory effect of E2 was mediated by GPER‐mediated nongenome pathway. First, we wanted to confirm the anti‐inflammatory effect of E2 postischemia. To do that, we have injected E2 (0.1 *μ*g/kg in 50 *μ*L DMSO) or 50 *μ*L DMSO only (vehicle control) 15 min after ischemia was induced and collected the serum at 3, 6, and 24 h. We have chosen this dosage of *β*‐estradiol, according to previous papers, to show the significant effect of *β*‐estradiol in protecting neurons from ischemia (Sudo et al. 1997). ELISA was then performed to measure the concentration of proinflammatory cytokine IL‐1*β* and TNF‐*α*.

We found that in the control group, both cytokines concentrations increased from 3 to 24 h postischemia. Compared to the control group, the IL‐1*β* concentration was reduced by the injection of E2 at 24 h (*n* = 5 rats, *P* < 0.0001, two‐way ANOVA with Sidak's multiple comparison test) and the TNF‐*α* concentration was reduced by the injection of E2 at 6 and 24 h (both 6 and 24 h, *P* < 0.0001), which confirmed that E2 has an inhibitory effect on inflammation and this effect has the fast onset within 6–24 h postischemia (Fig. 2A and B). To elucidate whether the anti‐inflammatory effect was due to GPER, we further injected the GPER antagonist G15 (in 50 *μ*L DMSO, 4.7 *μ*g/kg) along with the E2 and the GPER agonist G1 alone (in 50 *μ*L DMSO, 0.5 *μ*g/kg) after ischemia was induced. Compared with the control group, G1 reduced serum concentration of IL‐1*β* (3 h, *P* = 0.0036, 6 h, *P* = 0.0002, 24 h, *P* < 0.0001) and TNF‐*α* (3 h, *P* = 0.0420, 6 h & 24 h, *P* < 0.0001). G15 abolished the inhibitory effect of E2 on serum IL‐1*β* (3 h, *P* = 0.8048; 6 h, *P* = 0.6572, 24 h, *P* = 0.1682) and TNF‐*α* concentration (3 h, *P* = 0.9456, 6 h, *P* = 0.5393, 24 h, *P* = 0.2636), suggesting that E2 induced anti‐inflammatory effect was mediated by GPER (Fig. 2A and B) (Dennis et al. 2011; Jang et al. 2013).

### G1 and *β*‐Estradiol were able to inhibit the LPSinduced inflammatory effect in primary microglia culture

After establishing that GPER mediates the anti‐inflammatory effect of estradiol, we want to know whether this effect was mediated by GPER expressed on microglia as previously detected. We have established the technique of purifying microglia from rat brain tissue and maintaining the primary microglia culture. We added lipopolysaccharide (LPS, 10 ng/mL) to activate microglia for 8 h and found that the secretion of IL‐1*β* and TNF‐*α* by microglia significantly increased (*n* = 10 culture plates, *P* < 0.0001, unpaired Student's *t*‐test) compared with the control group (adding PBS only). In addition, application of *β*‐estradiol (final concentration of 100 nM) together with LPS could rescue the increased secretion of IL‐1*β* and TNF‐*α*, which suggested that *β*‐estradiol could inhibit the inflammatory effect of microglia (*n* = 10, IL‐1*β*,* P* = 0.0017; TNF‐*α*,* P* < 0.0001, one‐way ANOVA and post hoc test). To test if this effect of *β*‐estradiol was due to GPER, we added G1 (final concentration of 5 nmol/L) only or G15 + E2 (final concentration of G15 is 50 nmol/L) to LPS‐induced microglia and found that G1 could mimic the effect of *β*‐estradiol (*n* = 10, IL‐1*β* & TNF‐*α*,* P* < 0.0001, one‐way ANOVA and post hoc test) while G15 abolished the inhibitory effect of *β*‐estradiol (*n* = 10, IL‐1*β*,* P* = 0.4973, TNF‐*α*,* P* = 0.1627, one‐way ANOVA and post hoc test), which suggested that *β*‐estradiol was targeting on the GPER receptor on microglia. To summarize, *β*‐estradiol was able to suppress the secretion of IL‐1*β* and TNF‐*α* of cultured microglia and this effect was mediated by GPER.

### Knockdown of GPER abolished the inhibitory effect of *β*‐estradiol in primary microglia culture

To further confirm that *β*‐estradiol was targeting on GPER expressed on microglia, we used siRNA knockdown technique to inhibit the expression of GPER in the primary microglia culture. After transfecting siRNA, we tested the expression of GPER by western blot, and found that the expression of GPER was reduced in the siRNA‐transfected cells while not in control (untransfected) and sham (random oligonucleotide) group (Fig. 4A). After that, we repeated the experiment described above at 8 h postischemia, and found that while in knockdown group, microglia was still able to increase the secretion of IL‐1*β* and TNF‐*α* upon LPS stimulation, *β*‐estradiol and G1 were not able to reduce the secretion of IL‐1*β* (*n* = 10 culture plates, *P* = 0.2330, one‐way ANOVA) and TNF‐*α* (*n* = 10, *P* = 0.1017, one‐way ANOVA). This result strongly suggested that *β*‐estradiol‐induced anti‐inflammatory effect on microglia was mediated by GPER.

## Discussion

In this study, we have used the rat global cerebral ischemia model to find that GPER was expressed on microglia in forebrain cortex after ischemia. We demonstrated that estradiol possessed anti‐inflammatory effects including suppressing the serum concentration of IL‐1*β* and TNF‐*α* after ischemic stroke in vivo and also in primary microglia culture. The anti‐inflammatory effect of estradiol was mimicked by the GPER agonist G1 and inhibited by the GPER antagonist G15 in vitro and in vivo. In addition, knocking down of GPER by siRNA could also abolish the anti‐inflammatory effect of estradiol in primary microglia culture. Collectively, our results have suggested that estradiolinduced‐anti‐inflammatory effect postischemia was mediated through GPER expressed on microglia.

Ischemic stroke, which results from neuron death due to a lack of blood flow in the brain, could cause serious morbidity and even mortality (Donnan et al. 2008). In China, the prevalence of stroke grows over time along with the prevalence of hypertension and diabetes as the living standard enhances (Liu et al. 2011). Current treatment of ischemic stroke is focused on removing the blood clot either chemically by thrombolysis using rt‐PA method or surgically by thrombectomy (Meyers et al. 2011). Although effective in rescuing neurons and preventing disability postischemia, these treatments are limited to only a few hours after stroke. To find new treatment that are not limited to prompt timing, ischemia cascade after stroke has been intensively studied, which includes a lack of ATP supply, the excitotoxicity by glutamate, overload calcium inside neuron causing the neuronal death, and the prolonged inflammation after reperfusion and so on (Auriel and Bornstein 2010).

Recently, there is growing recognition of the importance of the prolonged inflammatory process postischemia including activating microglia cells, secreting proinflammatory cytokines, and recruiting blood inflammatory cells (Jin et al. 2010; Xing et al. 2012). This inflammation process, including the proinflammatory cytokines produced by activated microglia such as IL‐1*β*, IL‐6, and TNF‐*α* was shown to be detrimental to neuronal recovery after ischemia (Aloisi 2001; Nakajima and Kohsaka 2001). On the other hand, some immune mediators such as the anti‐inflammatory cytokine IL‐10 and TNF‐*β* could provide neuroprotection and reduce infarct size postischemic stroke (Amantea et al. 2014). During the inflammatory process, microglia activation is the earliest stage which also plays a central role in recruiting other blood inflammatory cells to cross the blood–brain barrier (Yenari et al. 2006; Pun et al. 2009). Inhibition of microglia activation could protect the integrity of blood–brain barrier, thus protect the brain from ischemic stroke (Yenari et al. 2006). Collectively, we think it is crucial to modulate the inflammatory effect of microglia to improve neuron survival postischemic stroke. Microglia, on the other hand, could also be beneficial and neuroprotective in the brain by releasing neurotrophic and anti‐inflammatory molecules to support neuron survival (Lehrmann et al. 1998; Parada et al. 2013; Patel et al. 2013). It could also help to eliminate toxic molecules and invading pathogens to maintain a beneficial microenvironment for neurons (Streit 2002). Loss of microglia or overreactive microglia could both exacerbate existing neurodegenerative diseases (Streit and Xue 2009). Thus, it is very important to understand microglia function during different pathologic conditions and design appropriate treatments accordingly.

Epidemiological studies have shown that women in reproductive age showed much less possibility of stroke than men, and the sex difference weakens with age, suggesting a possible link of estradiol with prevention of stroke (Hurn and Macrae 2000). Estradiol was demonstrated to possess neuroprotective function including reducing neuron injury, enhancing neurogenesis and behavior performance after cerebral ischemia (Hurn and Brass 2003). However, whether GPER could mediate the neuroprotective function of estradiol is still controversial. Some studies found that GPER was only elevated in male rats but not in female rats after ischemic stroke (Broughton et al. 2013). Some studies showed that activation of GPER is detrimental in male rats, but beneficial in ovariectomized female rats after ischemic stroke with regard to the infarct size and neuronal deficit (Broughton et al. 2014). Also some studies showed that activation of GPER could improve cerebral microvascular function after hypoxia injury in both female and male rats (Murata et al. 2013). This inconsistency could be due to difference in animal models and drug applications. This lack of understanding has limited the application of estradiol and GPER modulation in treatment of ischemic stroke. Here, we demonstrated that GPER mediates the anti‐inflammatory effect of estradiol both in female rat global ischemic model and in vitro, which could help design more specific drugs targeting inflammation postischemia. One limitation of our study is that we have used the primary microglia culture obtained from neonatal brain, which could behave differently from adult brain. To fully understand the function of estradiol, further studies of GPER expressed on other cells inside the brain are needed (Hazell et al. 2009).

## Conclusion

Our studies have demonstrated that GPER was highly expressed in activated microglia after ischemia and estradiol as well as the specific GPER agonist G1 could reduce the secretion of proinflammatory cytokines including IL‐1*β* and TNF‐*α*, which the anti‐inflammatory effect of G1 and E2 were both abolished by the specific GPER antagonist G15. Our studies have suggested that GPER‐mediated E2 evoked anti‐inflammatory effect. Our studies could not only help us to understand the mechanism of microglia mediated inflammation postischemia, but also potentially support the development of more specific drugs to manage inflammation postischemic stroke.

## Conflict of Interest

The authors declare that they have no conflict of interest.
